# Structure-Based Protein Assembly Simulations Including Various Binding Sites and Conformations

**DOI:** 10.1021/acs.jcim.4c00212

**Published:** 2024-04-11

**Authors:** Luis J. Walter, Patrick K. Quoika, Martin Zacharias

**Affiliations:** Center for Functional Protein Assemblies, Technical University of Munich, Ernst-Otto-Fischer-Str. 8, Garching 85748, Germany

## Abstract

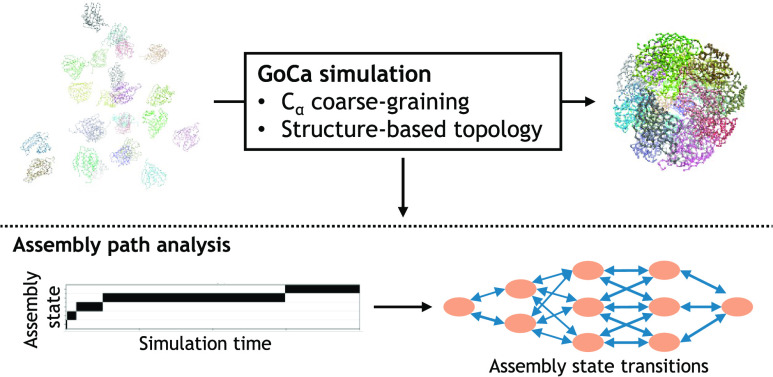

Many biological functions are mediated by large complexes formed by multiple proteins and other cellular macromolecules. Recent progress in experimental structure determination, as well as in integrative modeling and protein structure prediction using deep learning approaches, has resulted in a rapid increase in the number of solved multiprotein assemblies. However, the assembly process of large complexes from their components is much less well-studied. We introduce a rapid computational structure-based (SB) model, GoCa, that allows to follow the assembly process of large multiprotein complexes based on a known native structure. Beyond existing SB Go̅-type models, it distinguishes between intra- and intersubunit interactions, allowing us to include coupled folding and binding. It accounts automatically for the permutation of identical subunits in a complex and allows the definition of multiple minima (native) structures in the case of proteins that undergo global transitions during assembly. The model is successfully tested on several multiprotein complexes. The source code of the GoCa program including a tutorial is publicly available on Github: https://github.com/ZachariasLab/GoCa. We also provide a web source that allows users to quickly generate the necessary input files for a GoCa simulation: https://goca.t38webservices.nat.tum.de.

## 1 Introduction

Proteins are the workhorses of the cell and are responsible for a variety of functions, including enzymatic activity, signaling, substrate transport, or even mechanical work.^1,2^ Protein function is closely coupled to its structure, i.e., the molecular fold.^3^ Hence, resolving and understanding protein structure have been for several decades—and still is—of major interest to understand protein function.^4^ Frequently, not single proteins but assemblies of several proteins into multiprotein complexes are required to form functional units.^5^ Examples are multisubunit enzymes, chaperone complexes, the nuclear pore complex, or the proteasome for controlled degradation of proteins. Due to improvements in structure determination methods, especially of cryo-EM (electron microscopy of vitrified samples), the number of experimentally resolved multiprotein complexes has grown rapidly in recent years.^6^ In addition, recent breakthroughs in protein structure prediction based on deep learning approaches allow not only accurate prediction of single proteins but have also been extended to predict the structure protein complexes.^7−9^ However, the process of protein assembly formation from the individual protein subunits that may also involve protein refolding events is still difficult to study and poorly understood. Very little is known about intermediate states for most multiprotein complexes and if multiple pathways of association or a specific order of assembly events are required for correct multiprotein complex formation.

In principle, molecular dynamics (MD) simulations can be used to investigate the dynamics of protein structure and complex formation at atomic resolution and at high time resolution.^10^ The steady increase in computer performance allows for longer time scales and simulation of larger systems.^11^ Nevertheless, there are still limitations with respect to the system size, especially if slow processes are of interest. Computational models with lower spatial resolution and implicit solvation, termed coarse-grained (CG) models, offer an attractive route to extend system size and simulation time scale.^12−14^

One of the major challenges in the framework of CG simulations is the parametrization of the interactions between particles in the simulation, which is commonly expressed in terms of a force field. Most efforts to design CG force fields are based on trying to reproduce the results of atomistic simulations^12,13,15^ or to adjust parameters to reproduce experimental data.^16^ However, despite recent progress by employing artificial intelligence approaches,^17^ the design of a general CG force field that reproduces the native structure and dynamics of many different proteins has so far not been achieved. An alternative approach is the use of structure-based (SB) force fields.^18^ In this framework, the interactions in the simulations are modeled based on a known structure of the system such that folding simulations reproduce this known structure. The most well-known SB model is the Go̅-model, named after Nobuhiro Go̅,^19^ which involves purely repulsive interactions between all non-native contacts and attractive interactions only between contacts found in the native protein structure. Hence, by design, the native structure is the global minimum of the force field. Of course, such a force field is not universal, but parameters are different for each folded protein. The intention of simulations with SB models is not to predict the native structure but to answer more fundamental questions about the pathway of structure formation or how promoting non-native interactions may disturb the structure formation process. Go̅-type models have been successfully used to gain an understanding of folding processes, especially of small proteins.^20,21^ Furthermore, SB force fields have been adapted to investigate the structure of protein complexes.^22,23^ When studying multiprotein complexes that include copies of identical subunits, one needs to account for the possible permutations of such subunits in SB simulation approaches. Recently, the eGo approach,^23^ which combines SB terms with a standard atomistic force field description and accounts for such permutation effects, allowed to simulate the process of amyloid formation.

Here, we introduce a new setup of a Go-type model to investigate the assembly process of protein complexes consisting of an arbitrary number of subunits, termed GoCa. In particular, we adapted a backbone C_α_-based Go̅-type approach for single protein domains to be used with protein–protein complexes. The model distinguishes between subunit-internal and intersubunit interactions, enabling the investigation of simultaneous protein folding and protein association (coupled folding and binding). Furthermore, free permutation of identical subunits, for example, in homomultimeric complexes, is possible. In addition, the implementation allows not only one native structure as an attractor but also the definition of several metastable conformations (e.g., representing, e.g. open and closed protein states). Our implementation yields structural and topology input to be used with the GROMACS software,^24^ an established, well-optimized, and free simulation software. We also provide the user with a web service for convenient setup of the simulation system. GoCa allows the investigation of the assembly of arbitrary protein complexes out of the box if the assembled structure is provided (experimentally determined structure or predicted model). We describe several examples of how the approach can be used to study coupled protein folding and binding, assembly of a pentameric ring structure, and a multiprotein complex formed by the association of 24 subunits. The implications for studying the mechanism of assembly will be discussed.

## 2 Materials and Methods

### 2.1 GoCa Model Design

Below, we provide an in-depth explanation of our structure-based CG approach, i.e., the GoCa model. This model can be readily applied to any protein structure by using the GoCa program or web server. The GoCa program’s implementation details are outlined below. Generally, the model employs a well-established approach previously used for single protein chain folding to study the dynamics of the protein complexes. The amino acid coarse-graining allows the simulation of the dynamics of large multisubunit structures within a reasonable time. A SB force field is used to model the pseudobead interactions. The GoCa program generates simulation input files based on this model for the GROMACS simulation package. As a result, it is possible to use the optimized simulation algorithms of GROMACS for the simulation of the GoCa model. Running the GoCa program with a new protein structure requires minimal setup or configuration and is therefore straightforward.

The idea of the GoCa model is based on the work of Clementi et al.^25^ and the SMOG project.^26^ The GoCa program implements several features beyond regular SB models, making it especially suitable for simulating protein complexes. These features include support for multiple native conformations per protein chain, the merging of topologies for chains with identical sequences, and the possibility to modify the strength of different interaction surfaces. The details of these features are described in the following sections.

#### 2.1.1 CG Force Field

**Figure 1:**
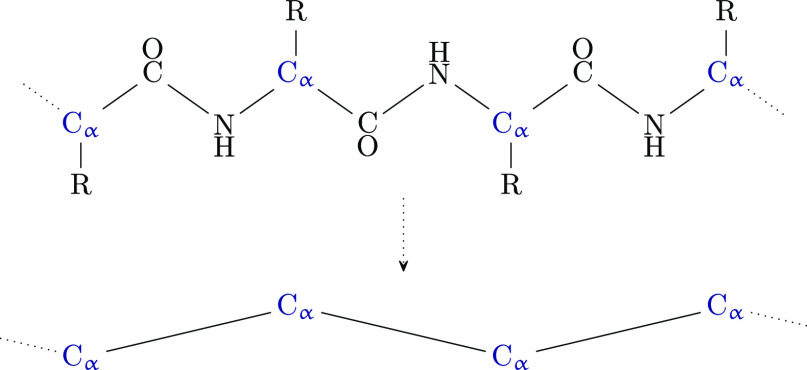
Schematic representation of C_α_ coarse-graining of a protein segment. The upper row shows an atomistic protein structure. R represents the amino acids’ side chains. The bottom depiction displays the same structure with CG C_α_ beads, as implemented in the GoCa model. The number of particles in the system is greatly reduced. Although all CG beads have the same general characteristics, such as size and mass, their interactions with each other are unique.

The GoCa model uses one pseudobead per amino acid. The principle purpose of combining multiple atoms into one pseudobead is to reduce the number of interactions in the system and thus increase simulation speed. The pseudo bead is placed at the location of the C_α_ atom. All pseudo beads have identical properties and only differ in their interactions. Characteristics such as residue size, mass, orientation, chirality, and atom number are ignored. Figure 1 visualizes the coarse-graining by comparing an all-atom peptide backbone segment to an equivalent chain in the GoCa model.

The GoCa model uses a SB approach to determine the interactions between the pseudobeads. The potential energy contributions are derived from the native, i.e., functional, conformation of the protein. The potential energy in our CG force field is calculated as the sum of different bonded and nonbonded contributions:
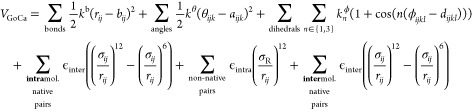
1Bonded interactions include harmonic bond length, harmonic bond angle, and periodic dihedral terms. Nonbonded interactions are modeled via a 12–6-Lennard–Jones potential. The GoCa model does not include explicit electrostatic interactions. However, these interactions are modeled implicitly because they affect the native conformation, which determines the overall SB force field.

Unlike atomistic force fields, the equilibrium distance and angle values for the interactions are calculated as the distances and angles in the native conformation of the target structure. The resulting force field is valid only for the target structure. However, the program can generate a new force field for any protein of interest based on an available native conformation. This simplified approach is justified by the folding-funnel theory, where the native conformation equals the conformation of minimal energy.^27^

Nonbonded interactions split into attractive and repulsive contributions. Amino acids close to each other in the native conformation are defined as native pairs and interact attractively. An atom-dependent cutoff determines whether two amino acids are close enough to be considered to be a native pair. In contrast, amino acid pairs that are not defined as native pairs interact repulsively (line 5 in eq 1). While other studies with a similar model use a plain C_α_–C_α_ distance cutoff, the GoCa model uses a more sophisticated approach. By applying a Van der Waals interaction-based cutoff^28^ for all-atom pairs between two amino acids, other native pair filter methods are not required. The GoCa model differentiates between inter- and intramolecular native pairs (lines 4 and 6 in eq 1).

The sum of all bonds includes all pseudobead pairs (*i*, *j*) with a direct peptide bond in the atomistic structure. In eq 1, the parameter *b*_*ij*_ represents the equilibrium distance between the C_α_ atoms from the native conformation. Similarly, the sum over all angles includes all pseudobead groups (*i*, *j*, *k*) with direct bonds between the pairs (*i*, *j*) and (*j*, *k*) in the atomistic reference. Parameter *a*_*ijk*_ denotes the equilibrium value for this bond angle from the native conformation. Moreover, the sum over dihedrals includes all groups (*i*, *j*, *k*, *l*) for which the amino acid pairs (*i*, *j*), (*j*, *k*), and (*k*, *l*) are each bound via a peptide bond. The dihedral is the angle between the two planes spanned by (*i*, *j*, and *k*) and (*j*, *k*, and *l*), respectively. The GoCa model uses two periodic dihedral contributions, one with multiplicity *n* = 1 and one with *n* = 3. Each contribution uses a different energy prefactor, *k*_*n*_^ϕ^. The equilibrium value *d*_*ijkl*_ for both dihedral terms is derived from the native conformation. The dihedral potential also ensures that structures do not refold into the mirrored native conformation since dihedrals of mirrored conformations have opposite signs. Similar to the bond–length interaction, parameter σ_*ij*_ is based on the equilibrium distance between particles *i* and *j*. Moreover, σ_R_ is derived from the radius of the repelling beads. The equilibrium distance or radius is divided by  to obtain the zero-crossing values σ. The default repulsive radius for all pseudobeads is 4 Å. As mentioned above, the general design of the potential is based on work by Clementi et al.^25^ However, here, we use a 12–6-Lennard–Jones potential instead of a 10–12-Lennard–Jones potential. The current version of GROMACS supports only the 12–6 variant for Lennard–Jones potentials. Work by Ferguson and Kollman^29^ suggests that, with appropriate parameters, both types of potentials give similar energies and structures. Since the energies of the GoCa model are rough approximations, the effect of differences between the two types of Lennard–Jones potentials for nonbonded interactions is considered negligible.

#### 2.1.2 Energy Units

All terms in the SB potential (given in eq 1) use force constant *k* or ϵ. These constants are equal for all interactions of the same type. This implies that the interaction strength only depends on the energy scaling parameter and not on the actual interaction strength of the atomistic amino acid interactions. This design choice makes GoCa model topologies easy to understand and parametrize. However, this property also makes comparing energies to real-world values challenging. We define all *k* parameters as multiples of general energy unit ϵ. As a result, several system properties depend on the value of this energy unit. These include, for example, the temperature and the length of the simulation time step. Therefore, calculations of these properties give results in so-called reduced units. They must always be regarded together with the chosen value for ϵ. Although theoretical conversion equations between reduced and real-world units exist,^30,31^ they are not suitable for the GoCa model. This is caused by the simple interaction design of the GoCa model, with fixed force constants per interaction type. Consequently, the results from GoCa model simulations are rather qualitative than quantitative. In the rest of this document, we will denote reduced temperature and time values with the units κ and τ, respectively.

#### 2.1.3 Merging of Native Conformations and Chains

The GoCa program can combine multiple chains and native conformations into a single topology. We implement this through a process called chain merging, which is described in this section. The program uses two approaches to combine topologies. The first applies to systems with multiple subunits that have identical amino acid sequences and similar native conformations. In this case, the GoCa program can merge the native conformations of the subunits into a single topology. Because their conformations are similar, their intramolecular force fields are also similar. Consequently, the resulting topology is valid for all merged chains. If such a complex with identical subunits is simulated without merged topologies, then permutations of the identical subunits are not possible. All subunits would interact attractively only with chains that are direct neighbors in the native conformation. In contrast, if the topologies are merged, then the subunits can assemble with any permutation. This behavior resembles reality with indistinguishable subunits much better than a regular SB model.

The second approach merges topologies of chains with distinct native conformations. This method is helpful in two cases: First, it allows for the combination of the topologies of multiple subunits of the same protein complex with identical sequences but different native conformations. Second, the user can provide multiple native conformations for a single chain via the input structure file in the GoCa program. In both cases, the resulting force field has multiple minima, one for each native conformation. Therefore, the structure can switch between the different conformations during the simulation. We achieve this by combining angle and dihedral potential contributions from multiple conformations via a tabulated minimum function. Native pair interactions are simply added for all native conformations. We justify this by the short range of Lennard–Jones interactions used for native pairs. Because of this short interaction range, native contacts that stabilize one conformation have little influence on the stability of another distinct conformation. Bond lengths are assumed to be constant for all of the conformations. Consequently, the topology can stabilize multiple conformations simultaneously.

The GoCa program automatically determines the chain merging approach based on the input files, the chain sequences, and a user-specified root-mean-square deviation cutoff parameter. The root-mean-square deviation cutoff is used to determine whether two native conformations are similar enough. It is also possible to combine both approaches. In this case, the program first merges the topologies of subunits with similar conformations and then combines the resulting topologies into a single topology with multiple stable conformations.

#### 2.1.4 Chain Interaction Groups

As mentioned above, in the GoCa model, all interactions of the same type have the same strength. Although this simple model design works well for many systems, one may want to adjust the interactions between different protein chains in some cases. One possible application is the influence of the assembly order for heteromeric complexes, e.g., based on previous computational or experimental insights. The GoCa program can automatically determine different interaction groups, i.e., clusters of native pairs that cause the total attractive interaction between two chain surfaces. In fact, the program calculates different types of interaction groups because this feature works with the chain merging described above. For example, a ring structure with several identical subunits has only one type of interaction group. In contrast, a homomeric structure with two stacked rings can have two or three interaction group types. The GoCa program allows for the modification of these interaction group types. Regarding the double-ring complex, one can adjust the interaction between the subunits within each ring to be stronger or weaker than the interaction between the two rings. The feature can be enabled via the configuration input file. The program then asks the user to specify the modification factors for each interaction group type.

### 2.2 GoCa Program Flow

**Figure 2:**
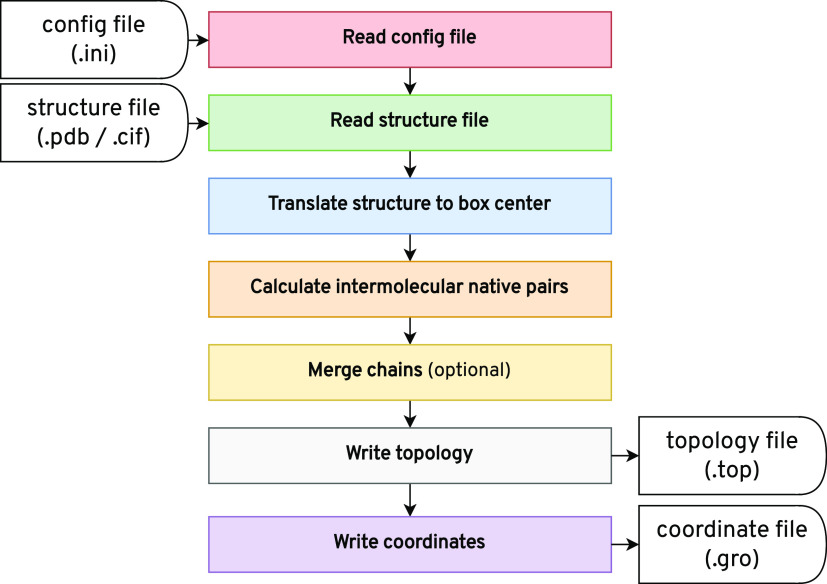
Flow diagram of the GoCa program. The process can be divided into seven program steps. The input to the program is a configuration and a structure file. It generates a topology and a coordinate file as the output.

We implemented the GoCa program in C++. Figure 2 shows the general program flow. First, the program reads the configuration and structure files. It generates pseudobead representations and organizes them into chains and models. Different models of the same chain represent different native conformations. Next, the program calculates the native pairs between different protein chains via a two-step process. First, chain pairs are filtered by their distance. Then, the program checks atom distances between close protein chains to find native pairs. During the optional chain topology merging, chains with the same amino acid sequence are combined into a single chain, as described above. This allows the output of a single topology for all subunits with the same sequence. After processing the structure, the program generates the topology and coordinate files. The GoCa program includes several example systems that serve as test cases and demonstrate the program’s functionality. The source code of the GoCa program is publicly available on Github: https://github.com/ZachariasLab/GoCa. This repository also contains a tutorial notebook, which is a good starting point for working with the GoCa model.

### 2.3 Simulation Workflow

Below, we describe the general workflow for the simulation of a protein with the GoCa model. The workflow consists of five steps. The first step is to prepare the native conformation. One provides the native conformation to the GoCa program as a Protein Data Bank (PDB) entry or mmCIF file. Experimentally determined or computationally predicted structures (e.g., obtained from the PDB^32^) are typically used. Before the GoCa program is run, it can be necessary to modify the available structure. First, nonstandard amino acids have to be removed or modified. For example, post-translational modifications must be substituted by the corresponding standard amino acid. Moreover, inputs to the GoCa program typically contain hydrogen atoms. Although they are not directly relevant to the CG structure, the default configuration for the native pair calculation assumes that hydrogen atoms are present. If a structure does not contain hydrogen atoms, then we recommend adding them retrospectively. Otherwise, one should adjust the native pair calculation parameters. If the structure has more than one native conformation, then these must be combined into one structure file. Each conformation has to correspond to one so-called model of the structure.

Theoretically, GoCa can be run without additional configuration parameters. However, many setups require parameters that differ from the default settings. Section A provides a detailed description of all configuration options.

Afterward, the GoCa program or Web server can be used to generate the topology and coordinate files for the simulation. If the starting configuration for the simulation differs from that of the native conformation, one can run the GoCa program twice with two different input structures. This allows the use of a topology from the native conformation and a coordinate file from another conformation. For example, the assembly simulation of a structure is typically not started with the assembled system but with randomly placed disassembled subunits. GROMACS provides various tools to modify coordinate files. For example, the gmx-tool insert-molecules(33) places molecules inside the simulation box at random positions with random orientations. The number of protein chains in the topology file can be adjusted manually in the [molecules] section at the end of the topology file. Thus, it is also possible to add excess subunits to the simulation that are not present in the native conformation.

Finally, the simulation can be run with the GROMACS simulation program. An exemplary configuration for the GROMACS simulation is provided in Section B. We recommend a preceding energy minimization step if the system contains randomly placed protein chains. Some proteins require adjusted parameters to prevent simulation instabilities. Splitting the system into different temperature coupling groups is the most effective adjustment, especially for large systems. This approach results in a more even velocity distribution and, therefore, prevents instabilities. Additionally, dihedral contributions can cause instabilities if one of the two corresponding bond angles is close to 180°.^34^ Modifying the GoCa program configuration parameter angle-dihedral-cutoff can prevent such dihedral instabilities. If simulations are still unstable, then one should reduce the simulation time step.

### 2.4 Test Systems

**Figure 3:**
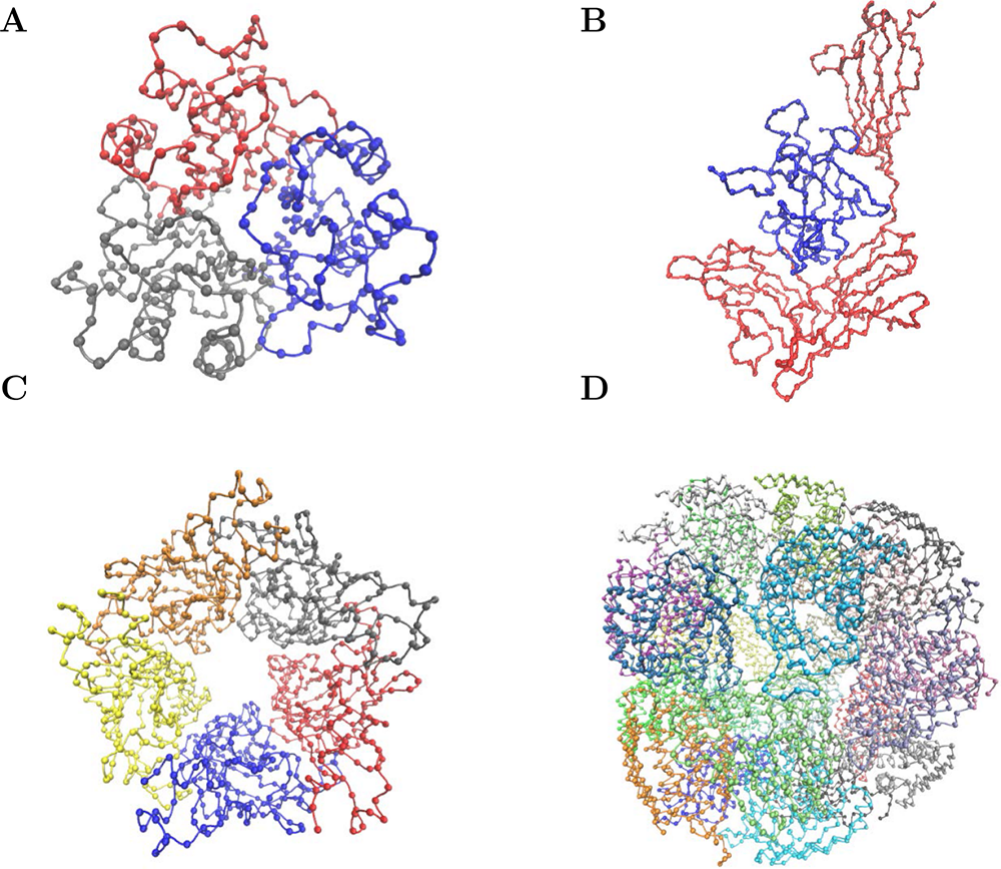
CG visualizations of our four test protein structures (created with VMD^35^) (A) Mammalian tumor-associated antigen UK114 (PDB: 1NQ3): homotrimeric complex isolated from goat liver. The trimer is stable in a crystal and in solution. Each subunit consists of 132 amino acids.^36^ (B) Interleukin-1 receptor with and without its antagonist (PDB: 1IRA and 1G0Y): The interleukin-1 receptor is a cytokine that is produced during inflammatory responses. It is the only cytokine with a known naturally occurring antagonist. Interleukin-1 (red) and its antagonist (blue) contain 309 and 145 amino acids, respectively.^37,38^ (C) Extracellular domain of α2 nicotinic acetylcholine receptor (PDB: 5FJV): Homopentameric ring complex with 208 amino acids per subunit.^39^ (D) Imidazoleglycerol-phosphate dehydratase (PDB: 6EZJ): Homo-24-meric capsid with 185 amino acids per chain. A key enzyme within the histidine biosynthesis pathway in plants and microorganisms.^40^

We used various protein systems to test the behavior of the GoCa model. In Section 3, we present results from four different systems. Figure 3 visualizes the CG structures. It shows the C_α_ beads and their covalent connections.

### 2.5 Trajectory Analysis

After handling periodic boundary conditions, the trajectory can provide insights into various properties of the simulated system. The GoCa model implementation aims to accelerate the simulation of the assembly processes of protein complexes. Therefore, the analysis focuses on the assembly states and pathways. The GoCa program provides different helper functions for Python to analyze multisubunit protein simulations. Those functions include, among others, methods to determine the intra- or intermolecular fraction of native contacts *Q*. Additional helper functions can generate graph representations for protein complexes. These graphs can be used to analyze the assembly pathways of the simulated protein complex. One graph representation per trajectory frame is calculated. In this representation, each protein chain corresponds to one graph node. Nodes are connected if the respective chains are bound. We use a moving average of the chain distance standard deviation to determine whether two chains are bound or not. Clustering these graph representations allows for analyzing the assembly as a discrete process with multiple intermediate steps. Further details are provided in Section D (SI).

## 3 Results

### 3.1 GoCa Model

As a first result, we present an overview of the GoCa model, which is an extended Go̅-like model specialized for protein complex assembly simulations. The model uses an amino acid-level CG model to reduce the number of particles in the system and therefore the computational complexity. Like regular Go̅ models, the GoCa model uses the native conformation of the simulated protein to determine the structure’s topology. However, the GoCa model has additional features that make it suitable for the simulation of protein complexes. First, it can combine multiple stable native conformations into a single topology. This feature allows the simulation of conformational changes during the assembly process. Second, the GoCa model can merge the topologies of multiple chains with identical sequences into a single topology. As a result, all of the identical chains in the system interact with each other in the same way. This enables a permutation-invariant assembly process for homomeric complexes. Third, modifying the interaction strength for specific protein chain pairs is possible. Adjusting the interaction strength makes integrating additional information about the actual interactions possible while maintaining the model’s simplicity. This is especially useful for the simulation of heteromeric complexes.

Combining these features makes the GoCa model suitable for the simulation of large protein complex assembly processes, which are not feasible with atomistic simulations. In the following subsections, we present four different examples of simulations with the GoCa model to elucidate possible applications.

### 3.2 Folding and Binding

**Figure 4:**
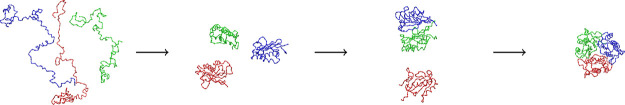
Snapshots from a GoCa model folding and assembly simulation of the protein UK114 (PDB: 1NQ3). The simulation starts with an unfolded configuration (first snapshot). All three subunits fold into their native conformations (second snapshot). Afterward, two subunits bind to form a dimer, while the third subunit remains unbound (third snapshot). Finally, the third subunit binds to the dimer to form the complete trimer structure (last snapshot).

As an initial example of a GoCa model simulation, we simulate the folding and assembly of a homotrimeric protein. The simulation uses the mammalian tumor-associated antigen UK114 (PDB: 1NQ3). Although the GoCa model focuses on assembling protein complexes, it is also suitable for simulating single-chain folding. Here, we combine both processes in a single simulation. To obtain a suitable starting configuration, we run an unfolding simulation at a high temperature of *T* = 200 κ for 4 nτ. We use the GoCa model configuration parameters ϵ_intra_ = ϵ_inter_ = 1.0. Afterward, the folding and assembly simulations run for 12 nτ at a temperature of *T* = 40 κ. In the beginning, all three subunits fold into their native conformation individually. Afterward, two subunits bind to form a dimer, while the third subunit remains unbound. Finally, the third subunit binds to the dimer to form the complete trimer structure. We show snapshots of these events from an example simulation in Figure 4.

Figure S1 shows the evolution of the assembly state, the fraction of native contacts, the root-mean-square deviation of the chains, and the total complex for this example simulation. After folding, the average root-mean-square deviation from the native conformation of the individual CG chains is approximately 2.2 Å. The example simulation was completed within 214 s on a regular desktop computer with a six-core processor and an NVIDIA GeForce RTX 2070 GPU. This simulation time is multiple orders of magnitude shorter than an atomistic simulation would require. Although this simple example does not provide significant new insights into the folding and assembly processes, it demonstrates the capabilities of the GoCa model. More complex systems with more subunits and more complex assembly pathways are possible.

### 3.3 Induced Fit Substrate Binding

We test the multiconformation behavior of the GoCa implementation with the interleukin-1 receptor protein with its antagonist. Two native conformations are used for the topologies of these two protein chains: The first contains the interleukin structure in its closed conformation (PDB: 1G0Y; this structure also includes an additional small peptide, which we removed for the simulation). In the second model, the antagonist is bound to the open interleukin conformation (PDB:1IRA).

**Figure 5:**
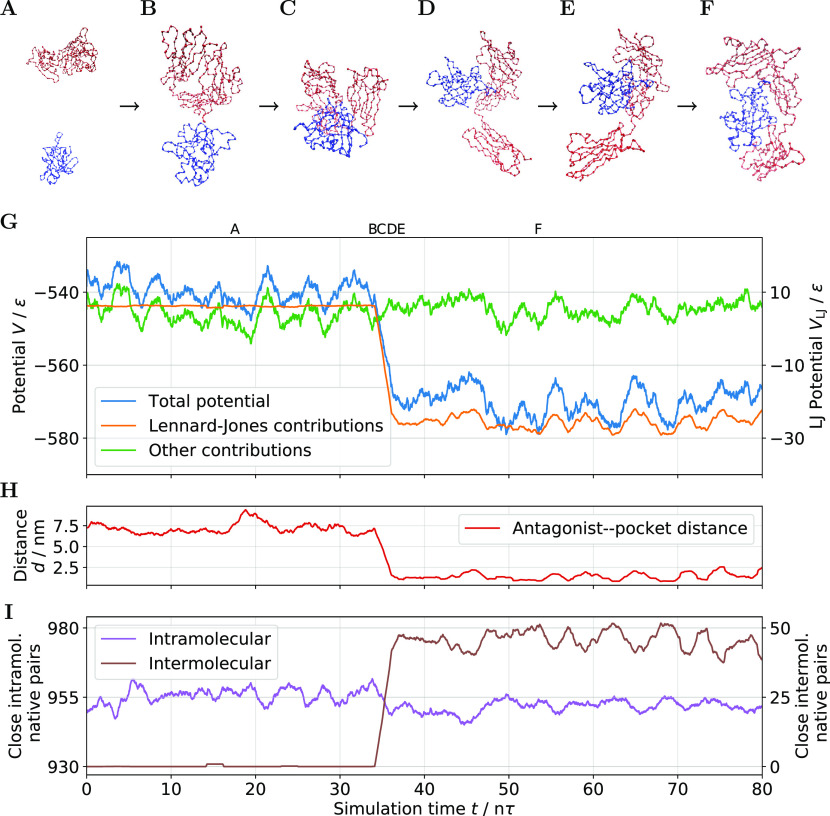
GoCa model binding simulation results of the interleukin-1 receptor and its natural antagonist (PDB: 1IRA and 1G0Y). (A–F) Simulation snapshots of the binding process. The GoCa model topology combines two stable native conformations. The simulation starts with the closed conformation (A). When the antagonist encounters interleukin, the interleukin conformation opens slightly (C). After the binding, the interleukin antagonist complex (F) remains stable until the end of the simulation. The scaling and viewing angles are different for each snapshot for better comprehensibility. (G) Evolution of the total potential, its native contact contribution, and the sum of other contributions during the binding event. (H) Distance between the antagonist and the binding location during the binding. (I) Evolution of the number of close inter- and intramolecular native contacts.

To evaluate the multiconformation behavior, the simulation starts with a separated configuration, i.e., the interleukin structure is closed and distant from the antagonist. During the simulation, both chains move separately through the simulation box. By chance, the antagonist comes close and binds to the interleukin. Until the binding event, interleukin stays in its closed conformation. It only starts to open due to the interaction with the antagonist chain. The complex remains bound until the end of the simulation. An energy analysis, visualized in Figure 5G, provides additional details about the binding process. Except for thermal fluctuations, the total potential energy remains relatively constant before and after the binding. However, during the binding, the potential energy decreases, i.e., the structure becomes more stable. The evaluation of the individual potential contributions explains this behavior. While the energy contribution from the bonded interactions remains relatively constant, the native pair contributions cause the potential to decrease. Even though some native pair contacts within the interleukin structure dissociate during binding, several new intermolecular contacts form between the two chains. The number of new native contacts exceeds the number of dissociated intramolecular native pairs. This behavior is also visible in Figure 5I. Generally, all angles and dihedrals that differ in the two conformations have two energy minima in their potential contribution. Nevertheless, their contribution to the transition between the conformations is insignificant. Instead, the inter- and intramolecular native pair interactions drive the conformational change. Nevertheless, tabulated angles and dihedrals avoid undue bias toward one conformation. Figure S2 shows the evolution of the root-mean-square deviation during the binding simulation. Using a 5-core CPU and an Nvidia GeForce RTX 2080 Ti GPU, the simulation achieved a performance of about 4.4 μτ/day.

### 3.4 Pentamer Assembly

This section provides the simulation results for a homopentameric ring complex. The assembly process of a five-subunit ring structure involves a significant number of intermediate states but remains simple enough for a detailed study. Here, we use the pentameric extracellular domain of the α2 nicotinic acetylcholine receptor (PDB:5FJV). Since all subunits of the structure have the same sequence, their permutation in the assembled structure is irrelevant. To simulate this permutation invariance, we used the chain merging feature of the GoCa program. To evaluate the assembly pathway, we simulate the pentamer assembly process 200 times with the GoCa model. Each simulation starts with all five subunits placed at random positions with random orientations inside the simulation box. The periodic simulation box has a side length of 18 nm. This value corresponds to a box padding of approximately 5 nm for the assembled structure. In general, the simulation box size affects the density and, therefore, the rate of random chain encounters required for chain association. Each simulation runs for 160 nτ at a temperature of *T* = 190 κ with ϵ_intra_ = 4.0 and ϵ_inter_ = 3.0. Using a three-core CPU and an Nvidia RTX A5000 GPU, each simulation requires an average simulation time of approximately 34 min. The final assembled structures have an average root-mean-square deviation from the native conformation of approximately 3.5 Å.

**Figure 6:**
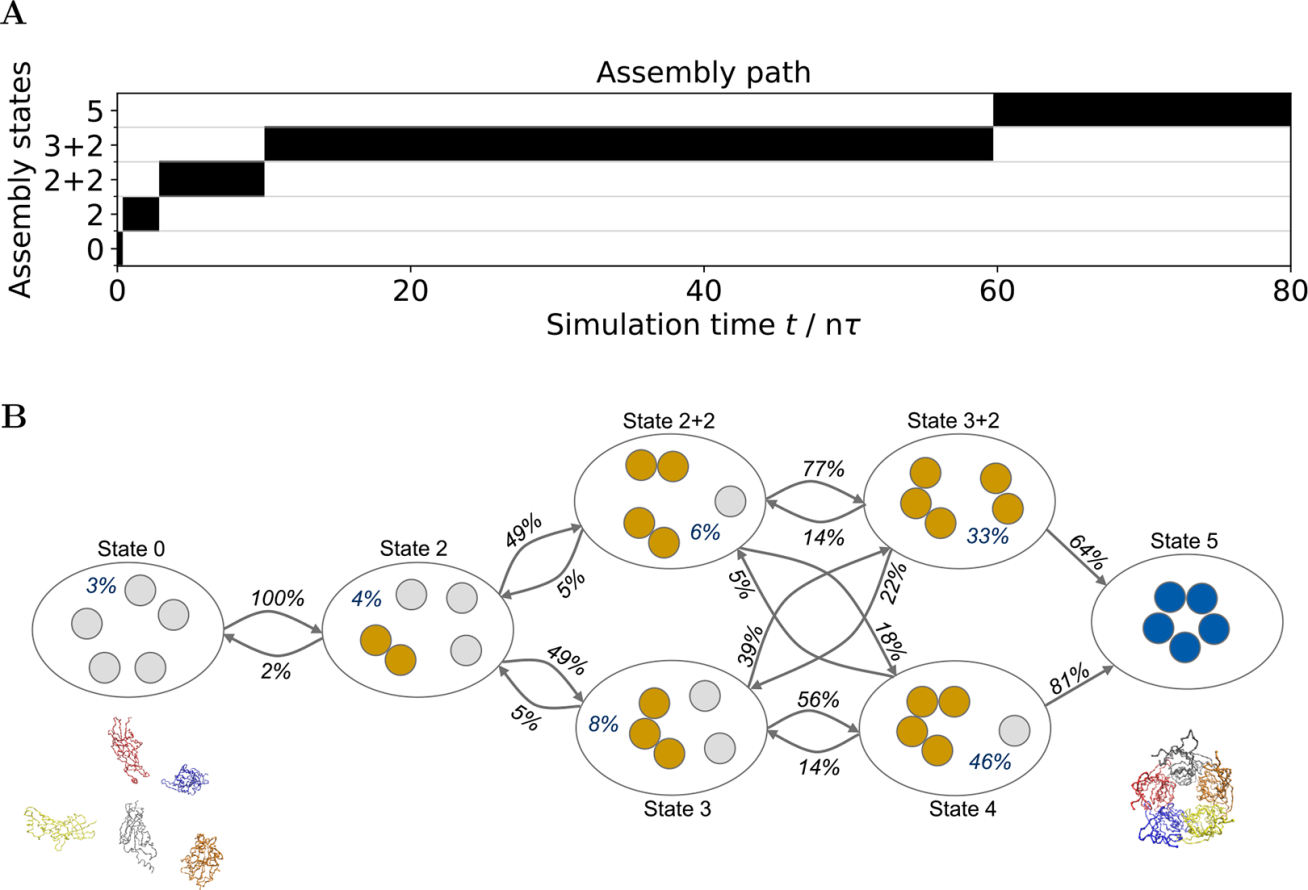
GoCa model assembly simulation results for the homopentameric α2 nicotinic acetylcholine receptor protein (PDB: 5FJV). There exist seven different discrete assembly states. (A) Visualization of the assembly path for an example simulation. This simulation spends very little time in state 0 (i.e., the completely disassembled state). Before full assembly, it stays in state 3 + 2 most of the time. (B) Visualization of assembly states and assembly state transitions from 200 simulations. Each ellipse illustrates one possible assembly state. Gray, brown, and blue circles represent disassembled, partially assembled, and fully assembled subunits. The figure also shows snapshots of the disassembled and fully assembled protein structure. Arrows with percentage values indicate outgoing transition probabilities, i.e., all outgoing transition probabilities per state add up to 100%. Multiplying the probabilities along an assembly path gives the total probability for that path. Percentage values per state indicate the expected amount of the total assembly time in which the system will remain in that state. Transition probabilities are computed with a lag time of one trajectory time step. This corresponds to a time of 4 pτ.

After the simulations, we clustered all trajectory time steps into different assembly states. The number and size of the assembled structures characterize each assembly state. For example, state 3 + 2 represents a conformation with a trimer and a dimer subassembly. Figure 6A shows the evolution of the assembly states for an example of the simulation. Initially, the simulation spends a very short time in state 0, i.e., the completely disassembled state. Then, two subunits bind to form a dimer (state 2). Afterward, a second dimer forms (state 2 + 2). The last free subunit binds to one of the dimers to form a trimer (state 3 + 2). Finally, the dimer and the trimer bind to form the complete pentamer (state 5). A combined evaluation of the assembly paths for all simulations provides additional insight into the overall assembly process. It allows the determination of transition rates and assembly state probabilities. Figure 6B visualizes these assembly states and their transitions. Transition probabilities are for outgoing transitions, i.e., the sum of all outgoing transition probabilities per state is 100%. 191 of the 200 pentamer simulations are successful assemblies, i.e., the simulations reach state 5 within the simulation time. The remaining nine simulations reach one of the states 4, 3 + 2, or 3. They would likely also assemble successfully with a greater simulation time. The most likely assembly path is 0 → 2 → 2 + 2 → 3 + 2 → 5 with a probability of about 24%. The second most likely assembly path is 0 → 2 → 3 → 4 → 5 which has a probability of 22%. Due to the stability of the fully assembled structure, the simulation data do not contain a reverse transition from state 5. Theoretically, this analysis also allows for transitions with more than one chain binding event per transition. However, with a short lag time of 4 pτ, the trajectories do not contain such a transition. The mean and median times until the complete assembly are 48.3 and 36.7 nτ, respectively. Figure S3 shows a histogram of the assembly times for all 191 successful simulations. Since atomistic simulations of this assembly process are not feasible, experimental confirmation of these results is required. However, experimental analysis of assembly path statistics is challenging.

### 3.5 24-mer Assembly

Although the assembly simulations of the pentameric structure in the last subsection are already beyond the capabilities of typical atomistic simulations, GoCa model simulations of much larger complexes are possible. To demonstrate this, we simulate the assembly of the protein imidazoleglycerol-phosphate dehydratase (PDB: 6EZJ). This protein forms a homomeric sphere structure with 24 subunits. We simulate the complex at a temperature *T* = 135 κ with ϵ_intra_ = 4.0 and ϵ_inter_ = 3.0. The density is approximately 477 nm^–3^, corresponding to a box padding of 4.5 nm for the assembled structure. To evaluate the assembly process, we perform 60 simulations with a duration of 1160 nτ. Each simulation starts with the 24 protein subunits randomly placed in the simulation box. We used different compute nodes with five processing units and one GPU for the simulations. For example, running one simulation on five cores of an Intel(R) Xeon(R) CPU E5-2640 v4 at 2.40 GHz and an NVIDIA GeForce GTX 1080 Ti took 15 h and 58 min. The average simulation wall clock time was approximately 17 h.

**Figure 7:**
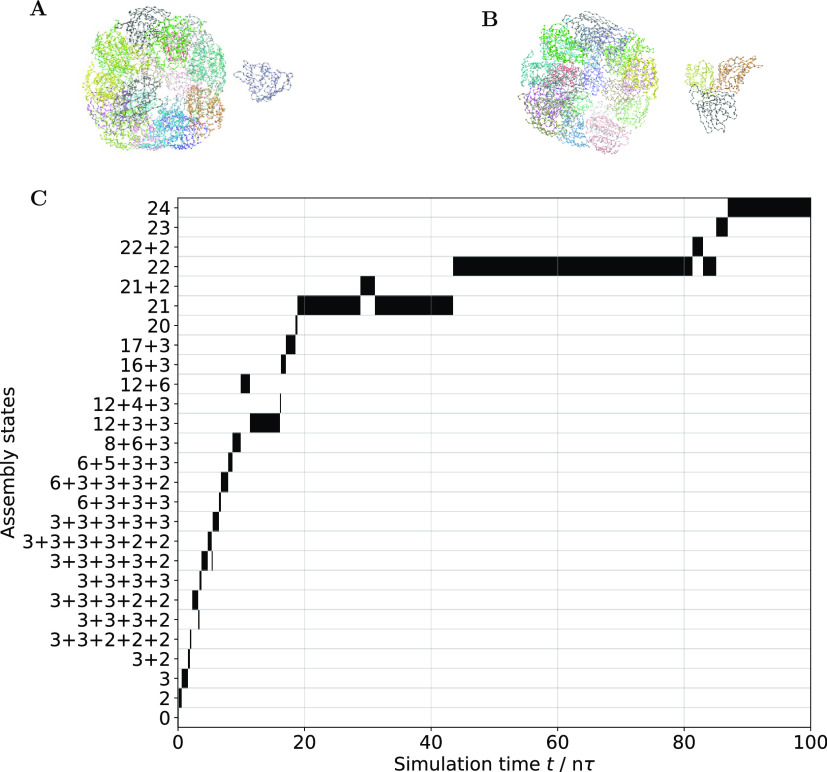
GoCa model assembly simulation results for the imidazoleglycerol-phosphate dehydratase protein (PDB: 6EZJ). (A) Snapshot of the protein structure in assembly state 23. (B) Snapshot of the protein structure in the 21 + 3 assembly state. (C) Assembly path diagram for an example simulation. The states on the vertical axis are sorted by the size and number of preassemblies. The diagram includes only assembly states that persist for at least 80 pτ. In the beginning, the subunits assemble much faster than they do at the end of the process.

After the simulations, the assembly states are calculated for all time steps of the trajectory, as described above. Of all 60 runs, 48 simulations assemble successfully within the simulation time. The average root-mean-square deviation of these 48 assemblies from their native structure is 2.8 Å with a standard deviation of 0.2 Å (see *D* for the calculation of chain-permutation invariant root-mean-square deviation values). The median assembly time for all successful assemblies is 354 nτ. Figure S4 shows a histogram of the assembly times. The assembly time corresponds to the time between the start of the simulation and the completion of the sphere structure. Since the assembled structure is relatively stable, we do not observe a dissociation of subunits afterward. Figure 7C visualizes the assembly path for an example of a simulation. Theoretically, an analysis similar to that shown for the pentamer in the previous subsection is possible. However, the number of possible intermediate assembly states is much larger due to the higher number of subunits. As a result, the variety of assembly pathways is enormous. This complicates the analysis and requires more samples to obtain reliable assembly path statistics. Considering the duration of an assembly simulation, it is costly to generate enough trajectories. Nevertheless, a smaller number of trajectories (e.g., from 60 simulations) allows various other statistics to be derived. Generally, the assembly process is faster at the beginning of the simulation. This behavior is due to a higher density of unbound subunits and more binding possibilities. Only a few unbound chains are available toward the end of the assembly process. Therefore, it takes more time for a successful subunit association to occur. Another notable behavior of the assembly process is the distribution of intermediate assemblies: Many subunits initially form trimer structures. As a result, other higher-order intermediate structures with multiples of three subunits are common. This behavior is also visible in the assembly path shown in Figure 7C. Moreover, this reproduces the experimental observations. The active 24-meric structure is known to assemble from inactive trimeric precursors.^41^

Since it is very challenging to sample the entire assembly exhaustively, only the last step of the process is analyzed in detail here. In almost all successful simulations, one of only two transitions leads to the completion of the protein structure. These transitions are 23 → 24 and 21 + 3 → 24. They occurred in 27 and 19 of all 48 successful simulations, respectively. Figure 7B, C shows snapshots of the two most common penultimate assembly states. This result also reflects the prevalence of trimeric substructures. In the remaining two successful assemblies, the second last assembly states are 12 + 12 and 18 + 6. Both cases have a low probability because the preassembled structures must be in a very particular shape. For example, in the case of 12 + 12, the “half spheres” need to be perfectly complementary. Otherwise, the binding of these substructures is not possible. In 10 out of 12 unsuccessful simulations, the system is in one of the two most common penultimate assembly states. Therefore, these structures would probably assemble successfully with a greater simulation time. The other two unsuccessful simulations end with 23 + 1 states in which the last subunit is bound to the structure in an incorrect position. Nevertheless, additional simulation time could lead to the dissociation of the incorrectly bound subunit followed by correct association. A similar behavior is visible in Figure 7C after 80 nτ.

The GoCa model simulations provide insight into the assembly process. Unfortunately, validating these results with atomistic simulations is challenging for multiple reasons: first, this system is considerably large (24 × 186 amino acids, diameter of the fully assembled state: 110 Å); second, the assembly process is slow; and last, the assembly process may occur on various pathways, as described above. Instead, experimental methods are required to confirm protein assembly pathways.^42^

## 4 Discussion

The GoCa simulation approach is an extended Go̅-type model that allows the study of the assembly process of multisubunit protein complexes. It is inspired by previous Go̅-type models based on the protein backbone^13,22,26^ and approaches that account for the permutation of multiple identical subunits.^23^ As with any SB approach, the intention is not to provide a universal approach to simulate protein folding and binding but to look at the process of assembly based on a known native complex.^12,25,26^ Beyond a standard Go̅ model, the separate treatment of intrasubunit and intersubunit interactions allows the investigation of coupled folding and binding upon complex formation.^43^ As a prerequisite to investigating homomultimeric complexes, or complexes with several identical copies of one protein, free permutation of identical subunits is automatically included.

Furthermore, the model allows the definition of multiple minima representing different possible (native) conformations for a given protein subunit. This can be useful for cases where a protein can adopt different open or closed conformations (depending, e.g., on the interaction with other protein partners) or for cases where identical domains in a multimeric structure have been resolved in slightly different conformations. It is important to note that since native pair interactions are the primary stabilizers even for multiple native conformations, a switch-like multimodel topology only works well if the conformations are sufficiently different. Otherwise, there is no significant energy difference between the conformations, and in such cases, the transitions between the native conformations are continuous movements between intermediate states. However, this behavior might even be desired for modeling flexible segments. In this case, combining multiple models that differ in the loose region causes a broadening of the minimum in the potential function.

Generally, it is important to emphasize that only qualitative and semiquantitative insight into the assembly process can be gained since physical parameters like temperature and simulation time are only defined relative to the attractive interaction strength. Hence, depending on the multiprotein complexes, an adjustment of a suitable simulation temperature might be necessary. Also, hydrodynamic interactions in a viscous solvent and how the subunit’s motions are hydrodynamically coupled—which may generally be relevant for assembly processes^44^—are not considered. Moreover, estimating kinetics from SB simulations is generally challenging.^45^ Nevertheless, the accumulation of intermediates in an assembly process gives qualitative insights into metastable configurations and steric barriers to assembly progression.

Combined with the GROMACS software,^24^ the GoCa simulation approach allows us to study assembly processes with dozens of subunits systematically with multiple simulations starting from many arbitrary starting placements even with limited computational resources. Several fundamental questions on the mechanism of multiprotein complex formation can be investigated systematically. It is possible to vary the interaction strength that controls the internal subunit flexibility or stabilizes the native complex geometry and to check the influence on the order and mechanism of the assembly process. Coupled folding and binding events may dramatically affect the order of formed intermediate states during assembly and can be investigated with the GoCa approach. By introducing various levels of non-native attractive interactions in future efforts, one can also systematically study the necessary balance relative to native interactions to achieve successful assembly formation. An interesting question that can be tackled is providing a surplus (or a deficit) of monomers of different types in the simulation box and checking the influence on the assembly mechanism in multiple simulations.

Another interesting application of GoCa is the assembly of large protein filaments such as actin. With our implementation, it is possible to assemble filaments of an arbitrary size. Based on the crystal structure of a short filament (e.g., the work of Oosterheert et al.^46^), GoCa may assemble filaments of any chosen length, depending on the number of monomers that are given. When we tested this approach, we found that adapting the strengths of longitudinal and transversal interfaces along the filament is necessary. This feature, which defines multiple binding sites (with varying binding strength) for proteins, may also help investigate the formation of heteromultimers. The interaction strength between different subunits may be adjusted independently, and the influence on the assembly process may be investigated. Adjusted interaction strength values could be, e.g., derived from experimental results. Although the GoCa program can automatically infer protein–protein interaction interfaces from the native conformation, the assembled complex structure must be available. Therefore, GoCa cannot predict interaction interfaces between subunits of a protein complex without a known assembled structure. However, other programs such as AlphaFold-Multimer^47^ can be used to predict the structure of the assembled complex, which can then be used as input for GoCa simulations.

The GoCa model utilizes an SB force field, which is straightforward to generate based on existing structures. Alternative approaches toward the parametrization of CG simulations are empirical force fields, multiscale models, machine learning-based force fields (potentially these follow a multiscale approach), or mixtures of such models, e.g., semiempirical force fields. These other approaches have proven successful. However, they are generally more challenging to parametrize. Below, we mention a few well-known examples, but clearly, there are many more successful applications. The complexes model, as introduced by Kim and Hummer,^48^ uses a C_α_-based empirical force field for the investigation of protein complexes.^49,50^ Their force field is based on the empirical amino acid interaction parameters of Miyazawa and Jernigan.^51,52^ In contrast to GoCa, proteins are modeled as rigid domains connected by flexible Gaussian linkers. Thus, the complexes model does not allow for arbitrary conformational changes. Another well-known example of semiempirical CG force fields is the MARTINI force field.^53,54^ Compared to GoCa, it has considerably higher resolution and is commonly used with explicit solvent. (An implicit solvent model, so-called dry MARTINI,^55^ also exists, though). Thus, it is computationally more demanding than that of GoCa. Multiscale approaches toward CG—such as described by Izvekov and Voth,^56^ Kamerlin et al.,^57^ or Hudait et al.,^58^ among others—are potentially more accurate than SB models. However, the parametrization of these models is much more demanding. Recently, Sahrmann et al.^59^ and Majewski et al.^60^ have investigated the possibility of using ML for the parametrization of CG simulations. These approaches look promising but, to the best of our knowledge, have not yet been tested extensively. Also, these approaches have general caveats, such as the demand for extensive training data, as described by Loose et al.^61^

The development of GoCa was inspired by the SMOG model, as published by Noel et al.,^26^ as described in Section 2.1. Compared with their work, we substantially extended the functionality by many features, as discussed above. Apart from the SMOG server, Lutz et al.^62^ have implemented the Python tool eSBMTools, which may be downloaded and run locally. It provides essentially the same models as the SMOG server with a few additional features, such as the possibility of manually adding arbitrary contacts. In addition to that, Scalone et al.^23^ have recently published a similar approach to GoCa, namely the multi-eGO model. This model is not purely SB since the bonded interactions are parametrized based on atomistic simulations (hybrid SB). The authors found that this approach yields better structural ensembles for disordered peptides than a purely SB approach. Furthermore, the multi-eGO model has near-atomic resolution, modeling all heavy atoms of the proteins. Thus, it is computationally considerably more demanding. The predecessor of multi-eGO, multi-GO^63^ (which is purely SB), originally used atomic resolution.

Our code is structured into different classes and therefore can be easily extended to include additional features. In the future, we plan to extend the current model to include nonprotein binding partners such as RNA and DNA or carbohydrate molecules.

## Data Availability

We provide a web-based tool for a convenient setup of GoCa simulations for multiprotein systems of interest: https://goca.t38webservices.nat.tum.de. The source code of the GoCa program is available on Github: https://github.com/ZachariasLab/GoCa. The repository also includes a tutorial with detailed instructions on the structure preparation, GoCa program execution, simulation, and analysis steps for an example system. The tutorial.ipynb notebook is included in the tutorial directory of the repository. MD trajectories, starting structures, force field topology files, and input/output files for all assembly simulations of the present study are deposited on Zenodo (Zenodo.org DOI 10.5281/zenodo.10869830).

## Supporting Information Available

(A) GoCa program configuration parameters, (B) example GROMACS configuration, (C) handling of periodic boundary conditions, (D) GoCa functions for trajectory analysis, and (E) additional result information (PDF)
