# Supplementary material for: Structure-Based Protein Assembly Simulations Including Various Binding Sites and Conformations
Source: J Chem Inf Model. 2024 Apr 11;64(8):3465–76. doi: 10.1021/acs.jcim.4c00212 (PMC11040733; doi:10.1021/acs.jcim.4c00212)
Supplement: Supplementary file 1 — ci4c00212_si_001.pdf [file ci4c00212_si_001.pdf]

# Supporting Information for Publication:

# Structure-based Protein Assembly Simulations Including Various Binding Sites and Conformations

Luis J. Walter[†], Patrick K. Quoika[†], and Martin Zacharias[†*]

[†] *Center for Functional Protein Assemblies, Technical University of Munich, Ernst-Otto-Fischer-Str. 8, Garching 85748, Germany*

E-mail: zacharias@tum.de

## Contents

**Supporting Information** **S2**

A GoCa Program Configuration Parameters . . . . . . . . . . . . . . . . . . . . . . S2

B Example GROMACS Configuration . . . . . . . . . . . . . . . . . . . . . . . . . S6

C Periodic Boundary Conditions . . . . . . . . . . . . . . . . . . . . . . . . . . . S7

D GoCa Functions for Trajectory Analysis . . . . . . . . . . . . . . . . . . . . . . S7

E Additional Results . . . . . . . . . . . . . . . . . . . . . . . . . . . . . . . . . S11

**References** **S14**

# Supporting Information

## A GoCa Program Configuration Parameters

Table S1: List of all configuration parameters including default values for the GoCa program.

**General**

`input` = input.pdb

PDB structure input file name (.pdb or .cif)

`topology` = output.top

Output topology file name

`coordinates` = output.gro

Output coordinate file

`type` = gromacs

Format for output files. Currently, only `gromacs` is supported.

`name` = Molecule

Molecule name for topology file

---

**Force field**

`epsilon` = 1.0

Energy unit for all **intra**molecular interactions ($\epsilon_{\text{intra}}$ in equation 1)

`bond-length` = 20000.0

Bond length energy multiplication factor ($k^{\text{b}}/\epsilon_{\text{intra}}$ in equation 1)

`bond-angle` = 40.0

Bond angle energy multiplication factor ($k^{\theta}/\epsilon_{\text{intra}}$ in equation 1)

`dihedral-1` = 1.0

First dihedral energy multiplication factor ($k_1^{\phi}/\epsilon_{\text{intra}}$ in equation 1)

`dihedral-2` = 0.5

Second dihedral energy multiplication factor ($k_3^{\phi}/\epsilon_{\text{intra}}$ in equation 1)

`intermolecular` = 1.0

Energy unit for all **inter**molecular interactions ($\epsilon_{\mathrm{inter}}$ in equation 1)

`radius` = 0.4

[nm] Radius for repulsive non-native pair LJ interactions ($\sigma_{\mathrm{R}}/\sqrt[6]{2}$ in equation 1)

`non-native-attraction` = no

{`yes`, `no`} Attractive LJ interaction for non-native pairs

---

**Model details**

`box-padding` = 3

[nm] Simulation box padding

`uniform-mass` = yes

{`yes`, `no`} Use uniform mass or original amino acid mass

`atomic-vdw-distance` = 0.05

[nm] Maximum atomistic VdW sphere distance for native pairs

`bead-vdw-distance` = 0.0

[nm] Maximum residue VdW sphere distance for native pairs
(only relevant if `include-bead-cutoff` is `yes`)

`include-bead-cutoff` = no

{`yes`, `no`} Use residue VdW radii for additional native pairs filtering

`atomic-cutoff` = 0.0

[nm] If provided use as a constant atomic cutoff for native pairs

`bead-cutoff` = 0.0

[nm] If provided use as a constant bead cutoff for additional
native pairs filtering (only relevant if `include-bead-cutoff` is `yes`)

`exclude-h-for-cutoff` = no

{`yes`, `no`} Exclude hydrogen atoms during native pair calculations

`excluded-number` = 3

Exclude non-bonding interactions between atoms that are no

further than this number of bonds away

`angle-dihedral-cutoff` = 155

[°] Ignore dihedrals if one of the two bond angles which share beads with the dihedral is bigger than this cutoff

`varying-interactions` = no

{`yes`, `no`} Ask interactively for factors to modify intermolecular interaction group strength

---

**Multi model**

`model-indices` = 1

Comma-separated model indices (starting from 1) in case of a multi-model structure input file with multiple native conformations

`table-directory` = tables

Directory to store tabulated potential files

`tabulation-points` = 100

Number of points for tabulated potential functions

`min-angle-dif` = 8.0

[°] Minimum bond angle difference for tabulation

`min-dihedral-dif` = 8.0

[°] Minimum dihedral angle difference for tabulation

---

**Other**

`save-config` = no

{`yes`, `no`} Write configuration to the topology output file

`save-nonbonded-info` = yes

{`yes`, `no`} Write info about intermolecular contacts to the topology file (distance, group, modification factor, possible interaction type)

`log-chains` = no

{`yes`, `no`} Print chain amino acid sequences to the console

`split-topology` =

If a file name is provided, chains are saved to separate `.itp` files

`cluster-cutoff` = 0.0

If larger than zero, chains are merged with this RMSD cutoff

`delete-other-models` = no

Delete all models except the first one for merged chains

## B Example GROMACS Configuration

```
integrator              = md
dt                      = 0.004      ; Timestep in ps
nsteps                  = 290000000  ; Number of simulation steps
nstenergy               = 1000       ; Energy output frequency
nstxout-compressed      = 1000       ; Coordinate ouput frequency
energygrps              = System     ; Energy groups
cutoff-scheme           = Verlet     ; Neighborsearching
nstlist                 = 20
pbc                     = xyz        ; Periodic boundaries
periodic-molecules      = no
verlet-buffer-tolerance = 0.005
rlist                   = 0.85
coulombtype             = Cut-off    ; Electrostatics treatment
rcoulomb                = 4.0        ; Not relevant, no charges
vdw-type                = Cut-off    ; VdW treatment
vdw-modifier            = Potential-shift-Verlet
rvdw                    = 4.0
table-extension         = 1
Tcoupl                  = v-rescale  ; Coupling algorithm
tc-grps                 = System
tau_t                   = 0.05       ; Time constant (ps)
ref_t                   = 135.00     ; Reference temperature
Pcoupl                  = no         ; Pressure coupling
constraints             = all-bonds  ; Bond constraining
constraint-algorithm    = LINCS
gen_vel                 = yes        ; Generate velocities
gen_temp                = 135.0
gen_seed                = -1
```

## C Periodic Boundary Conditions

After running the simulation, we recommend to process the trajectory files with the GROMACS tool `trjconv` to merge protein chains separated by the periodic boundary behavior. In the case of multi-subunit protein complexes, setting the `-pbc` option to `cluster` is useful. This periodicity treatment results in a correct resolution of the periodic boundary behavior for already assembled parts of the multi-subunit structure. However, this also introduces trajectory jumps for disassembled subunits. Nevertheless, the analysis typically focuses on the complex assembly instead of the trajectories of single subunits. If periodic boundary conditions for chains are handled automatically during the downstream analysis setting the `-pbc` option to `mol` is sufficient.

## D GoCa Functions for Trajectory Analysis

Additional Python functions are part of the GoCa program repository and help to analyze trajectories generated with the GoCa model. The most important functions are described here.

`gromacs_analysis.GromacsTrajectory`*`(trajectory_filename, coordinate_filename, topology_filename)`*

Constructs an `analysis.Trajectory` object from a GROMACS trajectory, native conformation coordinate file, and topology file. The topology file is only used to obtain defined native pairs. The library `mdtraj`[1] is used to read trajectory data and coordinate files.

`analysis.Trajectory.get_fraction_of_native_contacts_inside`*`(frame=None, chainId=None, offset=0.15,`*

*`sigmoid_factor=20, pbc=False)`*

Calculates the intramolecular fraction of native contacts $Q_{\mathrm{intra}}$. The ***`frame`*** parameter allows selecting a range of time steps or a single time step from the trajectory. If ***`chain_id`*** is specified, the result only includes contacts within the selected

chain. The cutoff threshold is equal to the native pair distances plus the *offset*. If *sigmoid_factor* is larger than zero, a sigmoid function is used to calculate $Q$:

$$Q = \big(1 + \exp(-\mathit{sigmoid\_factor} * (\mathit{cut\_off} - \mathit{distance}))\big)^{-1} \tag{1}$$

Otherwise, a hard cutoff is applied. If *pbc* is true, periodic boundary conditions are considered by using closest-image distances.

`analysis.Trajectory.get_fraction_of_native_contacts_between`*(frame=None, offset=0.15, sigmoid_factor=20, pbc=False)*

Calculates the intermolecular fraction of native contacts $Q_{\mathrm{inter}}$. All chains are included. The calculation of contacts and all parameters are as described for `analysis.Trajectory.get_fraction_of_native_contacts_inside`.

`analysis.Trajectory.get_fraction_of_native_contacts_chain_pairs`*(frame=None, offset=0.15, sigmoid_factor=20, pbc=False)*

Calculates $Q_{\mathrm{inter}}$ for all pairs of chains individually. The calculation of contacts and all parameters are as described for `analysis.Trajectory.get_fraction_of_native_contacts_inside`.

`analysis.Trajectory.get_chain_distance_map`*(pbc=False, overwrite_chain_slices=None)*

Calculates chain distance maps for the initial conformation (shape $N_{\mathrm{c}} \times N_{\mathrm{c}}$) and the trajectory (shape $N_{\mathrm{c}} \times N_{\mathrm{c}} \times N_{\mathrm{t}}$) with $N_{\mathrm{c}}$ the number of chains and $N_{\mathrm{t}}$ the number of time steps. The geometric center of each chain is used to calculate distances. A dictionary with chain indices as keys and bead-index slices as values can be provided as *overwrite_chain_slices* to modify bead index selections for individual chains. If *pbc* is true, periodic boundary conditions for chain centers are considered by using

closest-image distances.

`analysis.Trajectory.get_chain_graph(rolling_window_size=30, std_cut_off=0.3, distance_cutoff_factor=0.5, single_distance_cutoff=1.2, fixed_distance_cutoff=None, overwrite_chain_slices=None, logging=True, show_distance_histogram=False, processes=None)`

Generates `networkx.Graph`[2] objects for trajectories of multi-subunit structures. Each node in the graph represents one chain. Two nodes are connected if their corresponding chains are estimated to be bound. A list of graphs, i.e., one graph object per time step, is returned. Chains are estimated to be bound if the distance standard deviation for *rolling_window_size* steps is below *std_cut_off* and their mean distance is below a threshold. The algorithm attempts to determine this threshold from a distance histogram for all possible chain pairs (if *show_distance_histogram* is true, this is histogram is shown in a plot). It tries to determine the distance of the closest and second-closest neighbors and uses *distance_cutoff_factor* to define the cutoff. A *distance_cutoff_factor* = 0.5 implies a cutoff in the center of the closest and second-closest neighbor groups. For some structures, the program fails to determine neighbor groups and it uses the average distance multiplied by *single_distance_cutoff*. If *fixed_distance_cutoff* is provided, this is used as a fixed cutoff. A dictionary with chain indices as keys and atom-index slices as values can be provided as *overwrite_chain_slices* to modify atom index selections for individual chains. The parameter *processes* sets the number of processes for parallel calculations. If this is unspecified, the process number is equal to the number of CPUs.

`analysis.Trajectory.get_chain_formations(graphs, threshold=100, filtering=0, logging=False)`

Calculates an assembly state for each graph in *graphs*. An assembly state is defined by the size of all graph components with more than one subunit. Assembly states that occur less than *threshold* times are ignored. Occurrences of states that are shorter

than *filtering* time steps are ignored or overwritten by the most prevalent neighboring assembly state. The result is returned as an $N_{\mathrm{states}} \times N_{\mathrm{t}}$ array of boolean values. Additionally, the names of each state are returned as an array of size $N_{\mathrm{states}}$.

`analysis.Trajectory.get_chain_formations_detailed(graphs, threshold=100, filtering=0, logging=False)`

Calculates an assembly state for each graph in *graphs* similar to `analysis.Trajectory.get_chain_formations`. However, assembly states are defined differently and contain more details. Each state is defined by the size of all graph components and the number of nodes per node degree. In addition to the two-dimensional state array and the name array, a string is returned that describes the structure of the name array entries.

`analysis.Trajectory.permutation_invariant_self_rmsd(frame, angle_steps_per_axis=30, overwrite_chain_slices)`

Calculates the chain-permutation invariant RMSD between the initial conformation of the structure and the conformation at the trajectory frame with index *frame*. The method tries different rotations of the structure to find the best chain permutation. Depending on the number of tested angles, this does not guarantee that the optimal permutation is found, especially for incomplete assemblies. Nevertheless, for complete assemblies, this should give an accurate result. The method tries angles on the spherical grid specified by *angle_steps_per_axis*.

# E Additional Results

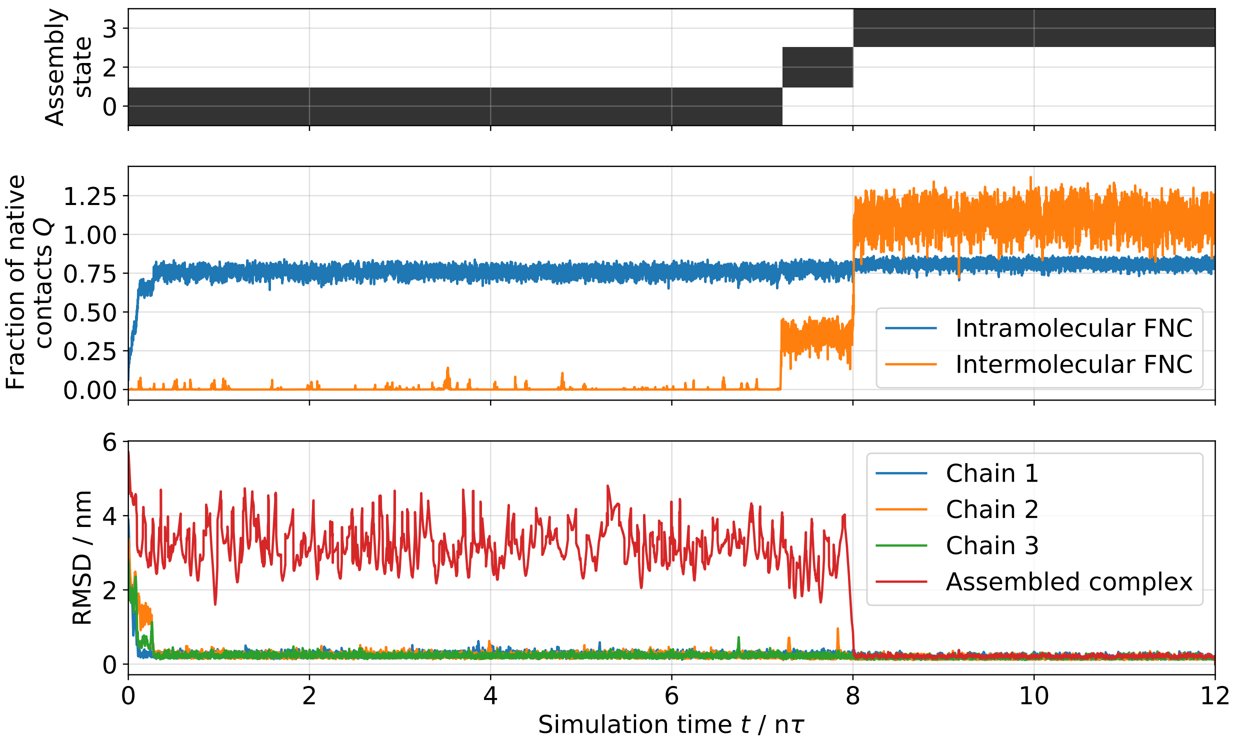


Figure S1: GoCa mode folding and assembly simulation of the of the protein UK114 (PDB: 1NQ3). The top diagram shows the evolution of the assembly state during the simulation. The middle diagram plots the intra- and intermolecular fraction of native contacts $Q$. Both $Q$ values are not equal to 1 in the assembled state due to thermal motion and the implementation of the counting algorithm. The bottom diagram visualizes the RMSD of the individual chains and the completely assembled complex. The simulation starts with a completely unfolded and disassembled configuration.

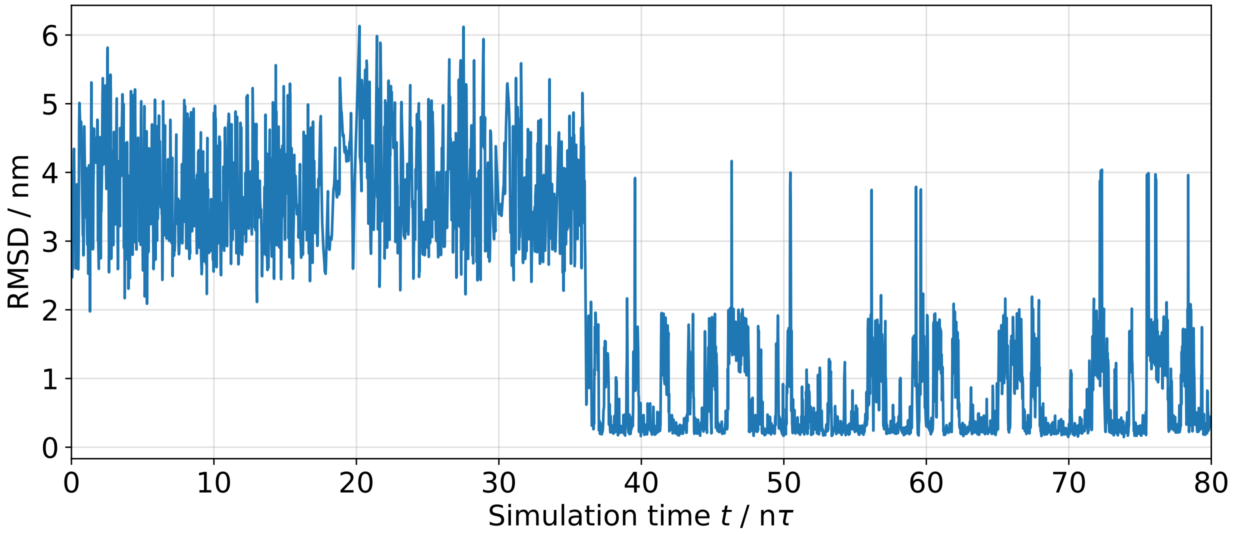


Figure S2: RMSD values for the GoCa model binding simulation of the Interleukin-1 receptor and its natural antagonist (PDB: 1IRA and 1G0Y). After the binding event, the RMSD values are significantly smaller than before, with some exceptions.

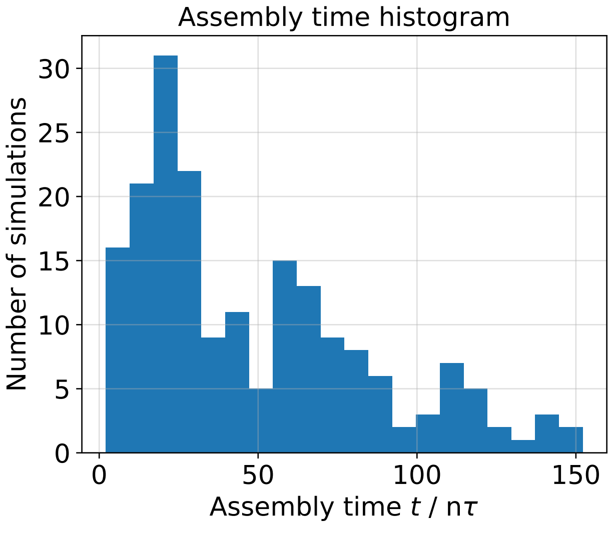


Figure S3: Histogram of the total assembly time for 191 successful assembly simulations of the homo-pentameric extracellular domain of the $\alpha 2$ nicotinic acetylcholine receptor (PDB: 5FJV). The remaining 9 out of 200 simulations did not successfully assemble until the end of the simulation after $160\,\mathrm{n}\tau$. The histogram uses 20 bins. The mean and median assembly times are $48.3\,\mathrm{n}\tau$ and $36.7\,\mathrm{n}\tau$, respectively.

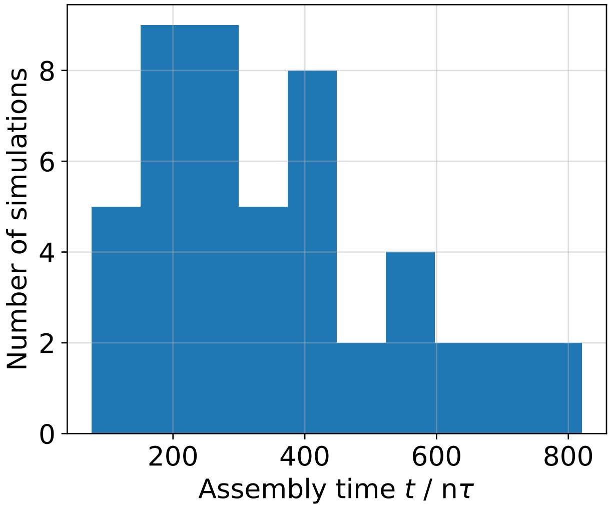


Figure S4: Histogram of the total assembly time for 48 successful assembly simulations of the Imidazoleglycerol-phosphate dehydratase protein. The remaining 12 out of 60 simulations did not successfully assemble until the end of the simulation after 1160 nτ. The histogram uses 10 bins. The mean and median assembly times are 354 nτ and 316 nτ, respectively.

# References


(1) McGibbon, R. T.; Beauchamp, K. A.; Harrigan, M. P.; Klein, C.; Swails, J. M.; Hernández, C. X.; Schwantes, C. R.; Wang, L.-P.; Lane, T. J.; Pande, V. S. MDTraj: A Modern Open Library for the Analysis of Molecular Dynamics Trajectories. *Biophys. J.* **2015**, *109*, 1528–1532.

(2) Hagberg, A. A.; Schult, D. A.; Swart, P. J. Exploring Network Structure, Dynamics, and Function using NetworkX. Proceedings of the 7th Python in Science Conference. Pasadena, CA USA, 2008; pp 11 – 15.
